# Effect of frozen-thawed embryo transfer with a poor-quality embryo and a good-quality embryo on pregnancy and neonatal outcomes

**DOI:** 10.1186/s12958-024-01194-x

**Published:** 2024-02-21

**Authors:** Cheng Zeng, Rui-Hui Lu, Xin Li, Sheng Wang, Yan-Rong Kuai, Qing Xue

**Affiliations:** Department of Obstetrics and Gynecology, Peking University First Hospital, Beijing, 100034 P. R. China

**Keywords:** Embryo quality, Double embryo transfer, Single good quality embryo transfer, Live birth rate, Neonatal outcomes

## Abstract

**Background:**

To evaluate the impact of embryo quality and quantity, specifically a poor quality embryo (PQE) in combination with a good quality embryo (GQE), by double embryo transfer (DET) on the live birth rate (LBR) and neonatal outcomes in patients undergoing frozen-thawed embryo transfer (FET) cycles.

**Methods:**

A study on a cohort of women who underwent a total of 1462 frozen-thawed cleavage or blastocyst embryo transfer cycles with autologous oocytes was conducted between January 2018 and December 2021. To compare the outcomes between single embryo transfer (SET) with a GQE and DET with a GQE and a PQE, propensity score matching (PSM) was applied to control for potential confounders, and a generalized estimating equation (GEE) model was used to determine the association between the effect of an additional PQE and the outcomes. Subgroup analysis was also performed for patients stratified by female age.

**Results:**

After PS matching, DET-GQE + PQE did not significantly alter the LBR (adjusted odds ratio [OR] 1.421, 95% CI 0.907–2.228) compared with SET-GQE in cleavage-stage embryo transfer but did increase the multiple birth rate (MBR, [OR] 3.917, 95% CI 1.189–12.911). However, in patients who underwent blastocyst-stage embryo transfer, adding a second PQE increased the live birth rate by 7.8% ([OR] 1.477, 95% CI 1.046–2.086) and the multiple birth rate by 19.6% ([OR] 28.355, 95% CI 3.926–204.790), and resulted in adverse neonatal outcomes. For patients who underwent cleavage-stage embryo transfer, transferring a PQE with a GQE led to a significant increase in the MBR ([OR] 4.724, 95% CI 1.121–19.913) in women under 35 years old but not in the LBR ([OR] 1.227, 95% CI 0.719–2.092). The increases in LBR and MBR for DET-GQE + PQE compared with SET-GQE in women older than 35 years were nonsignificant toward. For patients who underwent blastocyst-stage embryo transfer, DET-GQE + PQE had a greater LBR ([OR] 1.803, 95% CI 1.165–2.789), MBR ([OR] 24.185, 95% CI 3.285–178.062) and preterm birth rate (PBR, [OR] 4.092, 95% CI 1.153–14.518) than did SET-GQE in women under 35 years old, while no significant impact on the LBR ([OR] 1.053, 95% CI 0.589–1.884) or MBR (0% vs. 8.3%) was observed in women older than 35 years.

**Conclusions:**

The addition of a PQE has no significant benefit on the LBR but significantly increases the MBR in patients who underwent frozen-thawed cleavage-stage embryo transfer. However, for patients who underwent blastocyst-stage embryo transfer, DET-GQE + PQE resulted in an increase in both the LBR and MBR, which may lead to adverse neonatal outcomes. Thus, the benefits and risks of double blastocyst-stage embryo transfer should be balanced. In patients younger than 35 years, SET-GQE achieved satisfactory LBR either in cleavage-stage embryo transfer or blastocyst-stage embryo transfer, while DET-GQE + PQE resulted in a dramatically increased MBR. Considering the low LBR in women older than 35 years who underwent single cleavage-stage embryo transfer, selective single blastocyst-stage embryo transfer appears to be a more promising approach for reducing the risk of multiple live births and adverse neonatal outcomes.

## Background

As an effective procedure to allow for infertile patients to conceive, in vitro fertilization/intracytoplasmic sperm injection (IVF/ICSI) is used worldwide. Historically, many women undergoing IVF/ICSI have used two embryos during the transfer process to optimize reproductive outcomes. However, the increasing use of double embryo transfer (DET) has resulted in an inadvertently increased risk of multiple gestations [1, 2].

Multiple gestations are associated with significant complications and health risks for both mothers and infants [3]. Compared with singleton births, twins have a 4-fold increased risk of perinatal mortality, and for triplets, the risk increases 6-fold [4]. Twins from IVF/ICSI are at significantly greater risk for premature delivery, on average, 3 weeks earlier than singletons are, leading to a lower average birth weight of 850 grams (g) [5]. A twin pregnancy also increases the risk of obstetric complications, with high incidences of diabetes, hypertensive disorders, and placental abruption after IVF/ICSI [5]. Thus, the aim of assisted reproductive technology (ART) is to achieve healthy singleton gestation and reduce the occurrence of multiple gestations while maximizing the cumulative live birth rate [2].

A strongly recommended strategy for reducing the risk of multiple gestations is to consider the selective signal embryo transfer (SET) technique. Embryo quality, which is based on morphological parameters, is a major predictor of successful implantation and live birth and should be considered first for a successful SET [6]. A previous study assessing cleavage-stage embryos suggested that, compared with SET with a poor quality embryo (PQE), SET with a good quality embryo (GQE) is associated with a significantly lower ongoing pregnancy rate, depending on the number of GQEs available to transfer [7]. Apart from embryo quality, characteristics such as only one GQE available for cryopreservation, advanced age, and multiple failed previous cycles have been associated with poor pregnancy prognosis [1]. According to a recently published Cochrane meta-analysis, the live birth rate (LBR) may be lower in women who have undergone SET than in those who underwent DET [8]. As a result, despite the potentially higher multiple pregnancy rate, physicians generally tend to consider transferring an additional GQE to balance the risk of impairing the overall pregnancy rate when embryo quality declines or when patients exhibit characteristics associated with unfavorable pregnancy prognosis.

Although many studies have focused on differences in clinical outcomes between SET and DET with cleavage or blastocyst embryos [9, 10], only a few studies have compared SET with a GQE with DET with both GQE and PQE to minimize the risk of multiple gestation. Growing evidence suggests that crosstalk between the embryo and endometrium occurs during implantation, when the endometrium may be able to distinguish signals from competent embryos and developmentally abnormal embryos, and alter endometrial receptivity to protect mothers from the danger of abnormal pregnancies [11]. Thus, the transfer of a PQE with a GQE might send aberrant or harmful signals to the endometrium, which further leads to adverse reproductive outcomes. Previous studies have reported conflicting results when comparing IVF outcomes of SET with those of DET in fresh or frozen-thawed embryo transfer (FET) cycles. While some studies have shown that the addition of a PQE with a GQE results in a significantly lower implantation rate [12] or ongoing pregnancy [7], others have indicated that transferring a PQE along with a GQE does not diminish the likelihood of live birth [13–15]. Less control of confounding factors that may affect the LBR, such as age and ovarian reserve, ovarian stimulation protocols, and insemination methods, might lead to inconsistent results. Furthermore, to our knowledge, few studies have compared neonatal outcomes between DET with one GQE and an additional PQE and SET with one GQE.

Thus, the purpose of this study was to determine the impact of embryo quality and quantity, especially a PQE during DET with a GQE, on pregnancy and neonatal outcomes in patients undergoing FET cycles by using a propensity score matching (PSM) design and generalized estimating equation (GEE) models to control for possible confounding factors. Additionally, female patients were stratified according to age to investigate potential factors associated with poor prognosis.

## Methods

This was a retrospective observational cohort study of patients who underwent 1462 frozen-thawed cleavage or blastocyst embryo transfer cycles with autologous oocytes conducted at the reproductive medicine center of Peking University First Hospital (China) between January 2018 and December 2021. The study protocol was reviewed by the institutional ethics review board of Peking University First Hospital. The exclusion criteria were as follows: (a) had untreated hydrosalpinx prior to FET; (b) had blastocysts derived from vitrified oocytes or vitrified cleavages; (c) underwent mixed embryo transfer (ET) with a cleavage and a blastocyst embryo; (d) had known uterine anomalies, including intrauterine adhesion, septal uterine cavity, adenomyosis and fibroids with a diameter larger than 4 cm; (e) had uncontrolled endocrine and/or immune disorders; (f) had cycles with missing data; and (g) were lost to follow-up. Multiple cycles were performed for some patients in this study. Patients were grouped into a cleavage-stage embryo group or blastocyst group according to the developmental stage of the transferred embryos. Patients were subsequently divided into two groups according to the quantity and quality of the embryos. To compare the outcomes of SET with a GQE and those of DET with a GQE and a PQE, PSM was applied to control for potential confounders and selection biases. Moreover, a subgroup analysis was performed to explore the impact of transferring a second PQE with a GQE on live birth and neonatal outcomes; females were stratified by age, and women younger than 35 years and women 35 years old and older were included.

The details about the ovulation induction and IVF/ICSI stimulation protocols have been described in our previous studies [16, 17]. The initial and ongoing dosages were adjusted according to the patient’s age, ovarian response and previous IVF/ICSI procedure. Conventional IVF or ICSI was performed depending on the semen parameters and previous fertilization history. Normal fertilization was assessed approximately 16–18 h after insemination/injection. The embryos were cultured in G1/G2 media (Vitrolife, Denmark) for up to six days. Cleavage-stage embryos were classified as Grade I or II GQEs if they had seven to nine cells on Day 3, fewer than 20% anucleate fragments, equal-sized blastomeres in the majority of cells, and no multinucleation according to the ASEBIR embryo assessment criteria, with minor modifications [18]. Poor-quality cleavage-stage embryos (Grade III embryos) included those that had fewer than seven cells or more than nine cells on Day 3 and those that had seven to nine cells with more than 20% fragmentation. Blastocyst quality was graded on Day 5 or 6 according to Gardner’s classification system, and based on the degree of blastocyst expansion (Grade 1–6), inner cell mass (ICM, Grade A-C), and trophectoderm (TE) cells (Grade A-C) [19]. Good-quality blastocysts were graded as AA, AB, BA or BB with an expansion grade ≥ 3, while blastocysts with a lower quality than BB, such as BC and CB, with an expansion grade ≥ 3 on Day 5 or 6, were defined as PQEs. Cleavage-stage embryos with more than 50% fragmentation (Grade IV embryos) and blastocysts with poor morphological scores (≤ 4 CC) or low expansion grades (Grades 1–2) were not considered for vitrification or transfer.

After embryo grading, the embryos were cryopreserved by vitrification (KITAZATO, Japan) according to the manufacturer’s recommendations [20]. To thaw the embryos, they were directly immersed in thawing solution (TS) containing 1 mol/l sucrose (V900116, Sigma-Aldrich, Germany) at 37 °C for 1 min and subsequently incubated in each of the following solutions for 3 min: 0.5 mol/l sucrose, 0.25 mol/l sucrose and sucrose-free TS for stepwise cryoprotectant dilution [21]. The same vitrification and warming methods were employed throughout the whole study period. Endometrial preparation was performed as previously described [22]. (1) Natural cycle (NC): Patients with regular menses (21–37 days) would have a vaginal ultrasound examination on the 10th-12th day of the menstrual cycles to detect the leading follicle. Besides, the patients’ plasma levels of luteinizing hormone (LH), estradiol (E2) and progestin (P) were measured. ET was conducted on the day 3 (cleavage-stage ET) or the day 6 (blastocyst-stage ET) after ovulation. Luteal phase support (LPS) was commenced on the day of ET with oral dydrogesterone (Duphastone®, Abbott Biologicals, Holland) at a dose of 10 mg, twice daily, for 14 days. (2) hormone replacement treatment (HRT) cycle with or without gonadotropin-releasing hormone agonist (GnRHa) administration: Briefly, for patients with irregular menses (> 37 days), GnRHa injection was conducted during the early follicular phase using 3.75 mg triptorelin acetate (Dophereline®, Ipsen Pharma Biotech, France), HRT was performed 28–30 days after the GnRHa administration or on the 1st-5th day of the menstrual cycles using oral estradiol valerate (Progynova®, Delpharm Lille S.A.S., France) at a dose of 2–3 mg, three times a day. A vaginal ultrasound examination was performed 14 days later to detect the leading follicle and to measure the endometrial thickness. When the endometrial thickness reached at least 8 mm, ET was prepared. LPS was initiated on the day of ET using 10 mg dydrogesterone (Duphastone®, Abbott Biologicals, Holland), twice daily, along with 90 mg vaginal micronized progesterone (Crinone® 8%, Merck Serono, US) or 60mg progesterone injection (Zhejiang Xianju Pharmaceutical Co., China) once daily. One or two embryos were transferred according to the age and past history of the patients. If the pregnancy test was positive, LPS was continued until the 10th gestational week.

The primary outcome was LBR, defined as the number of live births after 28 weeks of gestation divided by the number of ET cycles. The secondary outcome was the multiple birth rate (MBR), which was defined as the live birth of multiple infants after 28 weeks of gestation divided by the total number of live births. Clinical pregnancy was confirmed by ultrasonographic visualization of the gestational sac 4–5 weeks after embryo transfer. Preterm birth was defined as a live birth before 37 weeks of gestation, and low birth weight was defined as a birth weight less than 2500 g.

### Statistical analysis

Data analyses were performed using the SPSS 22 package (SPSS, Inc., Chicago, IL, USA). The baseline characteristics were compared among the five groups. Categorical data are presented as numbers and percentages. Continuous variables are presented as the mean ± standard deviation (SD) and were tested using Student’s t test or nonparametric Mann–Whitney U test. To compare qualitative variables, chi-square and Fisher’s exact tests were used as indicated. All analyses of significance were 2-sided, and a value of *P* < 0.05 was used to indicate statistical significance.

PSM was applied to control for potential confounders and selection biases [23]. PSM was calculated using logistic regression based on potential variables related to the outcome by using the MatchIt package in R software (version 3.6.2). The variables included maternal age, maternal BMI, duration of infertility, serum anti-Mullerian hormone (AMH) concentration, antral follicle count (AFC), ovarian stimulation protocol, number of transplantable embryos, number of high-quality embryos, endometrial preparation protocol, endometrial thickness and day of blastocyst-stage embryo transfer. A 1:1 nearest neighbor matching method without replacement was conducted with a caliper width equal to 0.2 to match the data between SET with one GQE and DET with a GQE and a PQE.

Generalized estimating equation (GEE) models were constructed to evaluate the association between the effect of an additional PQE and patient outcomes by including patients who underwent multiple cycles and adjusting for propensity scores after matching [7]. To further verify the results, multivariate GEE models were generated using prematching data to adjust for the confounders mentioned above. Moreover, a subgroup analysis was also performed to explore the impact of transferring a second PQE with a GQE on IVF/ICSI outcomes stratified by age. Odds ratios (ORs) and 95% confidence intervals (95% CIs) were calculated from the model coefficients and their standard deviations.

## Results

In this study, a total of 1462 autologous frozen-thawed cleavage-stage embryo or blastocyst-stage embryo transfer cycles from 2018 to 2021 were evaluated. The patients’ overall baseline and treatment characteristics are presented in Tables 1 and 2 (left panel), stratified by embryo stage. As demonstrated in Table 1 (left panel), compared with DET-GQE + PQE patients before matching, patients who received single cleavage-stage embryo transfer with one GQE were younger and had a lower BMI, a higher level of serum AMH, a greater AFC, a lower amount of total Gn administration and more high-quality embryos, as these patients were likely to have a better ovarian reserve and cycles with a good outcome. Similarly, compared with patients who underwent double blastocyst-stage embryo transfer before matching, patients who underwent single good-quality blastocyst-stage embryo transfer were younger, had a higher serum AMH concentration, had a greater AFC and more transplantable and high-quality embryos, had received a lower total Gn administration, and adhered to the antagonist stimulation protocol and natural endometrial preparation protocol (Table 2 left panel). Thus, to further determine whether transferring a second PQE truly affects the outcomes of a single GQE, the primary analysis cohorts of the SET-GQE and DET-GQE + PQE cohorts were matched with the PS cohort based on potential variables related to the outcome. The results after PS matching are listed in Tables 1 and 2 (right panel), and the baseline characteristics were comparable between the two groups (*P* > 0.05).

**Table 1:** Baseline characteristics between single good-quality cleavage-stage embryo transfer and transfer of a second poor-quality embryo with a good-quality embryo before and after PS matching

Variable	Before matching			After matching		
	SET- GQE (*N* = 201)	DET- GQE + PQE (*N* = 421)	*P* value	SET- GQE (*N* = 169)	DET- GQE + PQE (*N* = 169)	*P* value
Maternal age (y)	32.6 ± 5.1	34.3 ± 5.2	**0.000**	33.1 ± 5.2	33.2 ± 5.2	0.900
Paternal age (y)	34.5 ± 5.9	35.9 ± 6.2	**0.006**	34.9 ± 6.0	35.2 ± 6.4	0.592
BMI (kg/m^2^)	21.9 ± 2.9	22.5 ± 3.4	**0.012**	22.0 ± 3.0	21.9 ± 3.0	0.610
Duration of infertility (y)	3.2 ± 2.5	3.3 ± 2.5	0.486	3.3 ± 2.6	3.4 ± 2.3	0.691
Type of infertility n (%)						
Primary	135 (67.2)	257 (61.0)	0.139	112 (66.3)	106 (62.7)	0.495
Secondary	66 (32.8)	164 (39.0)		57 (33.7)	63 (37.3)	
Basal FSH (IU/L)	9.8 ± 6.0	10.1 ± 6.3	0.589	10.1 ± 6.1	10.3 ± 6.1	0.720
AMH	3.8 ± 3.3	3.2 ± 3.0	**0.027**	3.5 ± 3.0	3.3 ± 2.8	0.457
AFC	13.3 ± 8.0	11.5 ± 7.3	**0.005**	12.6 ± 8.0	12.2 ± 7.4	0.631
Stimulation Protocol n (%)						
Agonist	54 (26.9)	137 (32.5)	0.154	44 (26.0)	51 (30.2)	0.612
Antagonist	114 (56.7)	204 (48.5)		94 (55.6)	85 (50.3)	
Others	33 (16.4)	80 (19.0)		31 (18.3)	33 (19.5)	
Total Gn (IU)	2364.6 ± 1080.2	2666.9 ± 1172.1	**0.002**	2386.0 ± 1117.0	2524.2 ± 1161.6	0.266
Duration of Gn (day)	9.6 ± 2.5	9.9 ± 2.6	0.189	9.5 ± 2.6	9.8 ± 2.7	0.360
Fertilization method n (%)						
IVF	120 (59.7)	224 (53.2)	0.128	96 (56.8)	100 (59.2)	0.659
ICSI	81 (40.3)	197 (46.8)		73 (43.2)	69 (40.8)	
Oocytes retrieved (n)	10.6 ± 7.5	9.6 ± 6.2	0.111	9.7 ± 7.0	9.9 ± 6.3	0.733
Number of transplantable embryos (n)	7.4 ± 5.6	6.6 ± 4.3	0.073	6.5 ± 5.0	6.7 ± 4.4	0.729
Number of top-quality embryos (n)	3.2 ± 2.4	2.1 ± 1.6	**0.000**	2.6 ± 2.1	2.6 ± 1.9	0.827
Endometrial thickness (mm)	9.9 ± 1.9	9.7 ± 2.0	0.264	9.8 ± 1.9	9.8 ± 2.0	0.939
Endometrial preparation protocol n (%)						
Nature	100 (49.8)	193 (45.8)	0.299	77 (45.6)	88 (52.1)	0.406
HRT	69 (34.3)	139 (33.0)		62 (36.7)	51 (30.2)	
HRT + GnRHa	32 (15.9)	89 (21.1)		30 (17.8)	30 (17.8)	

Values are expressed as n (%), percentage (%) or mean ± standard deviation (SD) unless otherwise stated. Variables presented in the part of statistical analysis were used for PS matching. *P*-values in bold depict statistical significance (*P* < 0.05) in comparison between SET-GQE and DET-GQE + PQE

*SET* Single embryo transfer, *DET* Double embryo transfer, *GQE* Good quality embryo, *PQE* Poor quality embryo, *BMI* Body mass index, *AMH* Anti-Müllerian hormone, *AFC* Antral Follicular Count, *Gn* Gonadotropin, *IVF* In vitro fertilization, *ICSI* Intracytoplasmic sperm injection, *HRT* Hormone replacement treatment, *GnRHa* Gonadotropin-releasing hormone agonist

**Table 2:** Baseline characteristics between single good-quality blastocyst transfer and transfer of a second poor-quality blastocyst with a good-quality blastocyst before and after PS matching

Variable	Before matching			After matching		
	SET- GQE (*N* = 523)	DET- GQE + PQE (*N* = 316)	*P* value	SET- GQE (*N* = 283)	DET- GQE + PQE (*N* = 283)	*P* value
Maternal age (y)	32.4 ± 4.2	33.4 ± 4.4	**0.001**	33.2 ± 4.3	33.1 ± 4.3	0.937
Paternal age (y)	34.1 ± 5.2	35.1 ± 5.5	**0.008**	34.9 ± 5.2	34.9 ± 5.5	0.994
BMI (kg/m^2^)	22.3 ± 3.3	22.8 ± 3.3	0.056	22.5 ± 3.3	22.7 ± 3.3	0.498
Duration of infertility (y)	3.0 ± 2.3	3.2 ± 2.3	0.285	3.1 ± 2.4	3.1 ± 2.3	0.990
Type of infertility n (%)						
Primary	343 (65.6)	202 (63.9)	0.626	173 (61.1)	185 (65.4)	0.295
Secondary	180 (34.4)	114 (36.1)		110 (38.9)	98 (34.6)	
Basal FSH (IU/L)	8.5 ± 4.8	9.2 ± 5.4	0.076	8.8 ± 5.6	9.2 ± 5.5	0.461
AMH	4.7 ± 3.8	3.8 ± 3.0	**0.000**	4.1 ± 3.1	3.9 ± 3.1	0.347
AFC	15.5 ± 7.4	13.9 ± 6.7	**0.001**	14.1 ± 6.9	14.1 ± 6.8	0.936
Stimulation Protocol n (%)						
Agonist	195 (37.3)	131 (41.5)	**0.041**	120 (42.4)	116 (41.0)	0.774
Antagonist	305 (58.3)	161 (50.9)		147 (51.9)	147 (51.9)	
Others	23 (4.4)	24 (7.6)		16 (5.7)	20 (7.1)	
Total Gn (IU)	2532.8 ± 975.0	2789.2 ± 1008.5	**0.000**	2716.5 ± 970.0	2736.0 ± 979.9	0.811
Duration of Gn (day)	10.1 ± 1.8	10.2 ± 2.3	0.504	10.4 ± 1.8	10.2 ± 2.3	0.488
Fertilization method n (%)						
IVF	352 (67.3)	217 (68.7)	0.681	184 (65.0)	193 (68.2)	0.422
ICSI	171 (32.7)	99 (31.3)		99 (35.0)	90 (31.8)	
Oocytes retrieved, n	13.4 ± 6.7	12.4 ± 6.0	**0.017**	12.9 ± 6.6	12.5 ± 5.9	0.348
Number of transplantable embryos (n)	9.9 ± 5.6	9.2 ± 4.5	**0.041**	9.5 ± 5.5	9.2 ± 4.3	0.455
Number of top-quality embryos (n)	4.2 ± 3.5	3.4 ± 2.9	**0.000**	3.9 ± 3.3	3.5 ± 2.9	0.118
Endometrial thickness (mm)	9.8 ± 1.9	9.8 ± 2.0	0.970	9.7 ± 1.9	9.7 ± 2.0	0.636
Endometrial preparation protocol n (%)						
Nature	288 (55.1)	132 (41.8)	**0.000**	140 (49.5)	130 (45.9)	0.626
HRT	167 (31.9)	102 (32.3)		88 (31.1)	90 (31.8)	
HRT + GnRHa	68 (13.0)	82 (25.9)		55 (19.4)	63 (22.3)	
Day of blastocyst transfer (%)						
Day 5	389 (74.4)	202 (63.9)	**0.001**	183 (64.7)	183 (64.7)	1.000
Day 6	134 (25.6)	114 (36.1)		100 (35.3)	100 (35.3)	

Values are expressed as n (%), percentage (%) or mean ± standard deviation (SD) unless otherwise stated. Variables presented in the part of statistical analysis were used for PS matching. *P*-values in bold depict statistical significance (*P* < 0.05) in comparison between SET-GQE and DET-GQE + PQE

*SET* Single embryo transfer, *DET* Double embryo transfer, *GQE* Good quality embryo, *PQE* Poor quality embryo, *BMI* Body mass index, *AMH* Anti-Müllerian hormone, *AFC* Antral Follicular Count, *Gn* Gonadotropin, *IVF* In vitro fertilization, *ICSI* Intracytoplasmic sperm injection, *HRT* Hormone replacement treatment, *GnRHa* Gonadotropin-releasing hormone agonist

Table 3 shows the pregnancy and neonatal outcomes of both groups before and after PS matching. When evaluating the outcomes between DET-GQE + PQE and SET-GQE in patients who underwent cleavage-stage embryo transfer, there were no significant differences in the clinical pregnancy rate (CPR), miscarriage rate or LBR, but DET-GQE + PQE was associated with a greater MBR (OR 3.917, 95% CI 1.189–12.911; *P* = 0.025) after matching. Moreover, compared with SET-GQE, DET-GQE + PQE resulted in similar neonatal outcomes but not significantly different outcomes in terms of preterm birth rate (PBR), birth height, birth weight or congenital anomalies in neonates.

**Table 3:** Clinical pregnancy and neonatal outcomes between patients who underwent single good-quality cleavage-stage embryo transfer and those who underwent transfer of a second poor-quality embryo with a good-quality embryo before and after PS matching

Variable	Cleavage-stage embryo transfer					
	Before matching			After matching		
	SET- GQE (*N* = 201)	DET- GQE + PQE (*N* = 421)	*P* value	SET- GQE (*N* = 169)	DET- GQE + PQE (*N* = 169)	*P* value
Implantation n (%)	78 (38.8)	208 (24.7)	**0.000**	63 (37.3)	93 (27.5)	**0.025**
OR (95%CI)	Reference	0.616 (0.428—0.886)	**0.009**	Reference	0.718 (0.450—1.145)	0.164
Clinical Pregnancy n (%)	76 (37.8)	167 (39.7)	0.657	61 (36.1)	74 (43.8)	0.183
OR (95%CI)	Reference	1.302 (0.885—1.914)	0.180	Reference	1.365 (0.876—2.127)	0.169
Live birth n (%)	63 (31.3)	134 (31.8)	0.903	53 (31.4)	66 (39.1)	0.172
OR (95%CI)	Reference	1.220 (0.808—1.841)	0.344	Reference	1.421 (0.907—2.228)	0.125
Multiple birth n (%)	5 (7.9)	29 (21.6)	**0.018**	4 (7.5)	16 (24.2)	**0.015**
OR (95%CI)	Reference	3.927 (1.273—12.113)	**0.017**	Reference	3.917 (1.189—12.911)	**0.025**
Miscarriage n (%)	13 (17.1)	33 (19.8)	0.624	8 (13.1)	8 (10.8)	0.680
OR (95%CI)	Reference	1.008 (0.510—1.991)	0.982	Reference	0.416 (0.061—2.833)	0.370
Preterm birth n (%)	7 (11.1)	20 (14.9)	0.468	6 (11.3)	10 (15.2)	0.543
OR (95%CI)	Reference	1.418 (0.555—3.625)	0.466	Reference	1.407 (0.459- 4.317)	0.550
Gestational age (weeks)	38.6 ± 1.9	38.2 ± 2.1	0.236	38. 6 ± 2.0	38.2 ± 1.9	0.225
OR (95%CI)	Reference	0.684 (0.382—1.223)	0.200	Reference	0.673 (0.347—1.306)	0.242
Birth height (mm)	49.3 ± 2.0	49.0 ± 2.2	0.283	49.3 ± 2.1	48.8 ± 2.1	0.186
OR (95%CI)	Reference	0.731 (0.418—1.278)	0.271	Reference	0.660 (0.341—1.277)	0.217
Birth weight (g)	3092.7 ± 563.0	3008.2 ± 595.2	0.319	3100.2 ± 588.7	2912.9 ± 555.6	0.059
OR (95%CI)	Reference	0.976 (0.928—1.026)	0.336	Reference	0.947 (0.890—1.008)	0.089
Low birth weight n (%)	11 (16.2)	32(19.6)	0.539	10 (17.5)	20 (24.4)	0.335
OR (95%CI)	Reference	1.673 (0.635—4.410)	0.298	Reference	1.688 (0.590—4.832)	0.329
Congenital anomalies n (%)	1 (1.5)	2 (1.2)	1.000	1 (1.8)	2 (2.4)	1.000
OR (95%CI)	-	-	-	Reference	1.279 (0.106- 15.369)	0.846

Values are expressed as n (%), percentage (%) or mean ± standard deviation (SD) unless otherwise stated. *P*-values in bold depict statistical significance (*P* < 0.05) in comparison between SET-GQE and DET-GQE + PQE. ORs were adjusted for variables presented in the part of statistical analysis using multivariate GEE model before matching. ORs after matching were adjusted for propensity score

*SET* Single embryo transfer, *DET* Double embryo transfer, *GQE* Good quality embryo, *PQE* Poor quality embryo, *OR* Odds ratio

However, in terms of blastocyst-stage embryo transfer, DET-GQE + PQE resulted in a significant increase in the risk of CPR (OR 1.579, 95% CI 1.123–2.221; *P* = 0.009), LBR (OR 1.477, 95% CI 1.046–2.086, *P* = 0.027) and MBR (OR 28.355, 95% CI 3.926–204.790, *P* = 0.001), as well as in the risk of PBR (OR 3.299, 95% CI 1.195–9.106, *P* = 0.021), when compared with SET-GQE (Table 4). Meanwhile, when transferring blastocyst-stage embryos, the gestational age (OR 0.572, 95% CI 0.345–0.948, *P* = 0.030) and birth weight (OR 0.943, 95% CI 0.903–0.984, *P* = 0.007) in patients with DET-GQE + PQE were significantly lower than those in patients with SET-GQE after matching. Adjusted ORs for congenital anomalies in women who underwent blastocyst-stage embryo transfer were not calculated because a multivariate GEE model was not applicable when the incidence of the variables was low.

**Table 4:** Clinical pregnancy and neonatal outcomes between patients who underwent single good-quality blastocyst transfer and those who underwent transfer of a second poor-quality blastocyst with a good-quality blastocyst before and after PS matching

Variable	Blastocyst transfer					
	Before matching			After matching		
	SET- GQE (*N* = 523)	DET- GQE + PQE (*N* = 316)	*P* value	SET- GQE (*N* = 283)	DET- GQE + PQE (*N* = 283)	*P* value
Implantation n (%)	274 (52.4)	227 (35.9)	**0.000**	137 (48.4)	207 (36.6)	**0.001**
OR (95%CI)	Reference	0.584 (0.454—0.752)	**0.000**	Reference	0.656 (0.485—0.886)	**0.006**
Clinical Pregnancy n (%)	269 (51.4)	180 (57.0)	0.120	134 (47.3)	163 (57.6)	**0.015**
OR (95%CI)	Reference	1.607 (1.180—2.188)	**0.003**	Reference	1.579 (1.123—2.221)	**0.009**
Live birth n (%)	213 (40.7)	140 (44.3)	0.309	104 (36.7)	126 (44.5)	0.060
OR (95%CI)	Reference	1.512 (1.109—2.060)	**0.009**	Reference	1.477 (1.046—2.086)	**0.027**
Multiple birth n (%)	2 (0.9)	27 (19.3)	**0.000**	1 (1.0)	26 (20.6)	**0.000**
OR (95%CI)	Reference	31.410 (6.749—146.186)	**0.000**	Reference	28.355 (3.926—204.790)	**0.001**
Miscarriage n (%)	56 (20.8)	40 (22.2)	0.722	30 (22.4)	37 (22.7)	0.949
OR (95%CI)	Reference	0.928 (0.570—1.513)	0.766	Reference	0.991 (0.573- 1.713)	0.974
Preterm birth n (%)	12 (5.6)	19 (13.6)	**0.010**	5(4.8)	18 (14.3)	**0.017**
OR (95%CI)	Reference	2.822 (1.247—6.384)	**0.013**	Reference	3.299 (1.195—9.106)	**0.021**
Gestational age (weeks)	39.0 ± 1.9	38.1 ± 2.4	**0.000**	38.9 ± 1.6	38.0 ± 2.5	**0.000**
OR (95%CI)	Reference	0.578 (0.364—0.916)	**0.020**	Reference	0.572 (0.345—0.948)	**0.030**
Birth height (mm)	49.7 ± 3.8	48.7 ± 3.6	**0.006**	49.5 ± 5.2	48.9 ± 3.2	0.233
OR (95%CI)	Reference	0.464 (0.251—0.858)	**0.014**	Reference	0.548 (0.282—1.068)	0.077
Birth weight (g)	3341.0 ± 517.2	3063.7 ± 615.8	**0.000**	3337.5 ± 561.9	3066.6 ± 617.8	**0.000**
OR (95%CI)	Reference	0.940 (0.907—0.975)	**0.001**	Reference	0.943 (0.903—0.984)	**0.007**
Low birth weight n (%)	13 (6.0)	23 (13.8)	**0.010**	8 (7.6)	21 (13.8)	0.123
OR (95%CI)	Reference	1.538 (0.601—3.933)	0.369	Reference	1.386 (0.526—3.656)	0.509
Congenital anomalies n (%)	0 (0)	1 (0.6)	0.437	0 (0)	1 (0.7)	1.000
OR (95%CI)	-	-	-	-	-	-

Values are expressed as n (%), percentage (%) or mean ± standard deviation (SD) unless otherwise stated. *P*-values in bold depict statistical significance (*P* < 0.05) in comparison between SET-GQE and DET-GQE + PQE. ORs were adjusted for variables presented in the part of statistical analysis using multivariate GEE model before matching. ORs after matching were adjusted for propensity score

*SET* Single embryo transfer, *DET* Double embryo transfer, *GQE* Good quality embryo, *PQE* Poor quality embryo, *OR* Odds ratio

The clinical outcomes of the patients who underwent cleavage-stage embryo transfer in the DET-GQE + PQE and SET-GQE cohorts stratified by age after matching are shown in Table 5. For women under 35 years old who underwent cleavage-stage embryo transfer, no significant differences in CPR or LBR were observed between the two groups. However, DET-GQE + PQE resulted in a significantly greater MBR (OR 4.724, 95% CI 1.121–19.913; *P* = 0.034) than did SET-GQE in women under 35 years old. For women 35 years of age and older, there were no significant differences in the CPR, LBR or MBR between the two groups. Additionally, similar neonatal outcomes were observed between the two groups, regardless of age (under or above 35 years). Furthermore, for patients ≥ 35 years of age who underwent transfer with a single good-quality cleavage-stage embryo, there was a robust trend toward a lower proportion of LBR (19.6% vs. 33.9%), but the difference did not reach clinical significance (*P* > 0.05).

**Table 5:** Clinical pregnancy and neonatal outcomes between patients who underwent single good-quality cleavage-stage embryo transfer and those who underwent transfer of a second poor-quality embryo with a good-quality embryo stratified by 35 years of age before and after PS matching

**Age < 35**	**Cleavage-stage embryo transfer**					
**Variable**	**Before matching**			**After matching**		
	SET- GQE (*N* = 143)	DET- GQE + PQE (*N* = 224)	*P* value	SET- GQE (*N* = 113)	DET- GQE + PQE (*N* = 113)	*P* value
Implantation n (%)	62 (43.4)	131 (29.2)	**0.002**	49 (43.4)	70 (31.0)	**0.024**
OR (95%CI)	Reference	0.546 (0.355—0.839)	**0.006**	Reference	0.577 (0.354—0.939)	**0.027**
Clinical Pregnancy n (%)	61 (42.7)	104 (46.4)	0.479	48 (42.5)	55 (48.7)	0.350
OR (95%CI)	Reference	1.210 (0.750—1.954)	0.434	Reference	1.264 (0.742—2.151)	0.389
Live birth n (%)	51 (35.7)	89 (39.7)	0.434	42 (37.2)	47 (41.6)	0.496
OR (95%CI)	Reference	1.137 (0.695—1.860)	0.609	Reference	1.227 (0.719—2.092)	0.454
Multiple birth n (%)	4 (7.8)	22 (24.7)	**0.013**	3 (7.1)	12 (25.5)	**0.021**
OR (95%CI)	Reference	4.421 (1.102—17.732)	**0.036**	Reference	4.724 (1.121—19.913)	**0.034**
Miscarriage n (%)	10 (16.4)	15 (14.4)	0.733	6 (12.5)	8 (14.5)	0.763
OR (95%CI)	Reference	0.921 (0.444—1.909)	0.824	Reference	0.418 (0.085—2.052)	0.283
Preterm birth n (%)	6 (11.8)	13 (14.6)	0.637	5 (11.9)	5 (10.6)	1.000
OR (95%CI)	Reference	1.232 (0.429—3.539)	0.698	Reference	0.890 (0.222—3.577)	0.870
Gestational age (weeks)	38.6 ± 1.7	38.1 ± 2.2	0.146	38.6 ± 1.9	38.3 ± 1.9	0.473
OR (95%CI)	Reference	0.682 (0.354—1.313)	0.252	Reference	0.813 (0.383—1.726)	0.590
Birth height (mm)	49.5 ± 2.1	49.1 ± 2.3	0.271	49.5 ± 2.2	49.2 ± 1.7	0.466
OR (95%CI)	Reference	0.673 (0.348—1.301)	0.239	Reference	0.818 (0.401—1.668)	0.580
Birth weight (g)	3116.2 ± 541.7	2977.0 ± 597.9	0.148	3128.7 ± 564.1	2974.6 ± 577.6	0.176
OR (95%CI)	Reference	0.964 (0.910—1.022)	0.218	Reference	0.965 (0.898—1.037)	0.336
Low birth weight n (%)	9 (16.4)	26 (23.4)	0.294	8 (17.8)	15 (25.4)	0.352
OR (95%CI)	Reference	1.867 (0.623—5.594)	0.265	Reference	1.722 (0.528—5.610)	0.367
Congenital anomalies n (%)	1 (1.8)	2 (1.8)	1.000	1 (2.2)	2 (3.4)	1.000
OR (95%CI)	-	-	-	Reference	1.384 (0.116- 16.454)	0.797
**Age ≥ 35**	**Cleavage-stage embryo transfer**					
**Variable**	**Before matching**			**After matching**		
	SET- GQE (*N* = 58)	DET- GQE + PQE (*N* = 197)	*P* value	SET- GQE (*N* = 56)	DET- GQE + PQE (*N* = 56)	*P* value
Implantation n (%)	16 (27.6)	75 (19.0)	0.129	14 (25.0)	23 (20.5)	0.510
OR (95%CI)	Reference	0.675 (0.320—1.421)	0.300	Reference	0.832 (0.372—1.858)	0.653
Clinical Pregnancy n (%)	15 (25.9)	63 (32.0)	0.374	13 (23.2)	19 (33.9)	0.209
OR (95%CI)	Reference	1.350 (0.630—2.893)	0.440	Reference	1.654 (0.712—3.839)	0.242
Live birth n (%)	12 (20.7)	45 (22.8)	0.729	11 (**19.6**)	19 (33.9)	0.088
OR (95%CI)	Reference	1.175 (0.512—2.698)	0.704	Reference	2.068 (0.867—4.933)	0.102
Multiple birth n (%)	1 (8.3)	7 (15.6)	0.863	1 (9.1)	4 (21.1)	0.626
OR (95%CI)	Reference	7.666 (0.481—122.201)	0.149	Reference	2.258 (0.212—24.020)	0.500
Miscarriage n (%)	3 (20.0)	18 (28.6)	0.727	2 (15.4)	0 (0.0)	0.157
OR (95%CI)	Reference	1.100 (0.232—5.209)	0.905	-	-	-
Preterm birth n (%)	1 (8.3)	7 (15.6)	0.863	1 (9.1)	5 (26.3)	0.372
OR (95%CI)	Reference	4.925 (0.034—704.376)	0.529	Reference	3.102 (0.323—29.829)	0.327
Gestational age (weeks)	38.4 ± 2.4	38.4 ± 1.9	0.938	38.4 ± 2.5	37.7 ± 1.8	0.345
OR (95%CI)	Reference	1.000 (0.372—2.687)	0.999	Reference	0.582 (0.146—2.324)	0.444
Birth height (mm)	48.8 ± 1.6	48.9 ± 2.2	0.954	48.8 ± 1.7	48.0 ± 2.6	0.341
OR (95%CI)	Reference	1.306 (0.464—3.674)	0.613	Reference	0.685 (0.210—2.234)	0.531
Birth weight (g)	2993.5 ± 660.5	3074.6 ± 589.6	0.666	2993.8 ± 689.9	2754.8 ± 470.1	0.234
OR (95%CI)	Reference	1.033 (0.926—1.152)	0.566	Reference	0.936 (0.825—1.063)	0.310
Low birth weight n (%)	2 (15.4)	6 (11.5)	1.000	2 (16.7)	5 (21.7)	1.000
OR (95%CI)	-	-	-	Reference	1.539 (0.134—17.640)	0.729
Congenital anomalies n (%)	0	0	-	0	0	-
OR (95%CI)	-	-	-	-	-	-

Values are expressed as n (%), percentage (%) or mean ± standard deviation (SD) unless otherwise stated. *P*-values in bold depict statistical significance (*P* < 0.05) in comparison between SET-GQE and DET-GQE + PQE. ORs were adjusted for variables presented in the part of statistical analysis using multivariate GEE model before matching. ORs after matching were adjusted for propensity score

*SET* Single embryo transfer, *DET* Double embryo transfer, *GQE* Good quality embryo, *PQE* Poor quality embryo, *OR* Odds ratio

When the analysis was expanded to patients who underwent blastocyst-stage embryo transfer (Table 6), for patients under 35 years old, DET-GQE + PQE resulted in a significantly greater CPR (OR 1.626, 95% CI 1.017–2.599, *P* = 0.042) and LBR (OR 1.803, 95% CI 1.165–2.789, *P* = 0.008) than did SET-GQE. However, transferring a PQE with a GQE resulted in a significant increase in MBR (OR 24.185, 95% CI 3.285–178.062, *P* = 0.002) and PBR (OR 4.092, 95% CI: 1.153–14.518, *P* = 0.029), which may have led to a trend toward lower gestational age and birth weight than those who underwent singleton good-quality blastocyst-stage embryo transfer. Among those patients 35 years of age and older, although the CPR (OR 2.282, 95% CI 1.458–3.571; *P* = 0.000) was greater in patients with DET-GQE + PQE, no significant differences in the LBR or MBR were observed (*P* > 0.05). In addition, the birth weight (OR = 0.923, 95% CI = 0.862–0.987, *P* = 0.020) of neonates was lower in patients who underwent DET-GQE + PQE than in patients who underwent embryo transfer with a single good-quality blastocyst.

**Table 6:** Clinical pregnancy and neonatal outcomes between patients who underwent single good-quality blastocyst transfer and those who underwent transfer of a second poor-quality blastocyst with a good-quality blastocyst before and after PS matching

**Age < 35**	**Blastocyst transfer**					
**Variable**	**Before matching**			**After matching**		
	SET- GQE (*N* = 379)	DET- GQE + PQE (*N* = 197)	*P* value	SET- GQE (*N* = 181)	DET- GQE + PQE (*N* = 181)	*P* value
Implantation n (%)	218 (57.5)	159 (40.4)	**0.000**	97 (53.6)	146 (40.3)	**0.003**
OR (95%CI)	Reference	0.567 (0.416—0.772)	**0.000**	Reference	0.654 (0.454—0.941)	**0.022**
Clinical Pregnancy n (%)	213 (56.2)	123 (62.4)	0.150	94 (51.9)	113 (62.4)	**0.044**
OR (95%CI)	Reference	1.607 (1.085—2.380)	**0.018**	Reference	1.626 (1.017—2.599)	**0.042**
Live birth n (%)	167 (44.1)	99 (50.3)	0.157	70 (38.7)	90 (49.7)	**0.034**
OR (95%CI)	Reference	1.674 (1.147—2.445)	**0.008**	Reference	1.803 (1.165—2.789)	**0.008**
Multiple birth n (%)	2 (1.2)	24 (24.2)	**0.000**	1 (1.4)	23 (25.6)	**0.000**
OR (95%CI)	Reference	31.641 (6.827—146.651)	**0.000**	Reference	24.185 (3.285—178.062)	**0.002**
Miscarriage n (%)	46 (21.6)	24 (19.5)	0.650	24 (25.5)	23 (20.4)	0.376
OR (95%CI)	Reference	0.748 (0.404—1.383)	0.355	Reference	0.713 (0.370—1.373)	0.312
Preterm birth n (%)	8 (4.8)	15 (15.2)	**0.004**	3 (4.3)	14 (15.6)	**0.022**
OR (95%CI)	Reference	3.441 (1.330—8.906)	**0.011**	Reference	4.092 (1.153—14.518)	**0.029**
Gestational age (weeks)	39.0 ± 2.0	38.1 ± 2.3	**0.000**	38.9 ± 1.8	38.0 ± 2.3	**0.005**
OR (95%CI)	Reference	0.566 (0.324—0.989)	**0.046**	Reference	0.573 (0.304—1.080)	0.085
Birth height (mm)	49.7 ± 4.3	48.6 ± 3.3	**0.016**	49.3 ± 6.3	48.9 ± 2.5	0.605
OR (95%CI)	Reference	0.389 (0.198—0.764)	**0.006**	Reference	0.578 (0.248—1.345)	0.203
Birth weight (g)	3330.3 ± 548.8	3053.9 ± 610.6	**0.000**	3300.0 ± 637.7	3067.4 ± 606.0	**0.014**
OR (95%CI)	Reference	0.937 (0.898—0.979)	**0.003**	Reference	0.951 (0.899—1.006)	0.078
Low birth weight n (%)	12 (7.1)	18 (14.6)	**0.036**	8 (11.3)	16 (14.2)	0.571
OR (95%CI)	Reference	1.530 (0.561—4.172)	0.406	Reference	0.957 (0.340—2.699)	0.957
Congenital anomalies n (%)	0 (0)	1 (0.8)	0.421	0 (0)	1 (0.9)	1.000
OR (95%CI)	-	-	-	-	-	-
**Age ≥ 35**	**Blastocyst transfer**					
**Variable**	**Before matching**			**After matching**		
	SET- GQE (*N* = 144)	DET- GQE + PQE (*N* = 119)	*P* value	SET- GQE (*N* = 102)	DET- GQE + PQE (*N* = 102)	*P* value
Implantation n (%)	56 (38.9)	68 (28.6)	**0.037**	40 (39.2)	61 (29.9)	0.102
OR (95%CI)	Reference	0.602 (0.380—0.954)	**0.031**	Reference	0.661 (0.396—1.103)	0.113
Clinical Pregnancy n (%)	56 (38.9)	57 (47.9)	0.142	40 (39.2)	50 (49.0)	0.159
OR (95%CI)	Reference	1.722 (1.046—2.835)	**0.032**	Reference	2.282 (1.458—3.571)	**0.000**
Live birth n (%)	46 (31.9)	41 (34.5)	0.667	34 (33.3)	36 (35.3)	0.768
OR (95%CI)	Reference	1.003 (0.582—1.729)	0.992	Reference	1.053 (0.589—1.884)	0.862
Multiple birth n (%)	0 (0)	3 (7.3)	0.101	0 (0)	3 (8.3)	0.240
OR (95%CI)	-	-	-	-	-	-
Miscarriage n (%)	10 (17.9)	16 (28.1)	0.197	6 (15.0)	14 (28.0)	0.140
OR (95%CI)	Reference	1.322 (0.455—3.839)	0.608	Reference	2.318 (0.794—6.771)	0.124
Preterm birth n (%)	4 (8.7)	4 (9.8)	1.000	2 (5.9)	4 (11.1)	0.674
OR (95%CI)	Reference	2.310 (0.295—18.101)	0.425	Reference	1.972 (0.352—11.040)	0.440
Gestational age (weeks)	38.9 ± 1.1	38.1 ± 2.8	0.105	38.9 ± 1.0	38.0 ± 3.0	0.063
OR (95%CI)	Reference	0.518 (0.281—0.955)	**0.035**	Reference	0.542 (0.224—1.311)	0.174
Birth height (mm)	49.9 ± 0.9	48.9 ± 4.4	0.128	50.0 ± 0.7	48.7 ± 4.7	0.103
OR (95%CI)	Reference	0.512 (0.185—1.422)	0.199	Reference	0.475 (0.132—1.709)	0.475
Birth weight (g)	3368.5 ± 383.2	3090.9 ± 636.5	**0.014**	3415.9 ± 351.0	3064.2 ± 658.9	**0.005**
OR (95%CI)	Reference	0.928 (0.869—0.992)	**0.029**	Reference	0.923 (0.862—0.987)	**0.020**
Low birth weight n (%)	1 (2.2)	5 (11.4)	0.107	0 (0)	5 (12.8)	0.057
OR (95%CI)	-	-	-	-	-	-
Congenital anomalies n (%)	0	0	-	0	0	-
OR (95%CI)	-	-	-	-	-	-

Values are expressed as n (%), percentage (%) or mean ± standard deviation (SD) unless otherwise stated. *P*-values in bold depict statistical significance (*P* < 0.05) in comparison between SET-GQE and DET-GQE + PQE. ORs were adjusted for variables presented in the part of statistical analysis using multivariate GEE model before matching. ORs after matching were adjusted for propensity score

*SET* Single embryo transfer, *DET* Double embryo transfer, *GQE* Good quality embryo, *PQE* Poor quality embryo, *OR* Odds ratio

## Discussion

In view of the results of this study, we demonstrated that the transfer of an additional PQE along with a GQE did not have a beneficial effect on clinical outcomes, as the addition of a PQE resulted in no significant difference in the LBR but increased the MBR for cleavage-stage embryo transfer. However, for patients who underwent blastocyst-stage embryo transfer, DET-GQE + PQE resulted in a notable increase in both the LBR and MBR, which may lead to adverse neonatal outcomes, such as more preterm births, younger gestational age and lower birth weight. Specifically, in patients younger than 35 years who underwent cleavage-stage embryo transfer, the addition of a PQE increased only the MBR in comparison with those who underwent single GQE transfer, but the additional PQE resulted in a significantly greater LBR and MBR in patients younger than 35 years with blastocyst-stage embryo transfer. Conversely, in patients aged 35 years and older, DET-GQE + PQE did not significantly differ from SET-GQE in terms of LBR or MBR, regardless of the embryo-stage. Although an increased LBR was observed in patients who underwent blastocyst-stage embryo transfer, the MBR increased dramatically, especially in patients younger than 35 years. In patients younger than 35 years, SET-GQE resulted in a satisfactory LBR either in cleavage-stage embryo transfer or blastocyst-stage embryo transfer, while DET-GQE + PQE resulted in a dramatically increased MBR. Thus, in order to minimize the risk of multiple live births and adverse neonatal outcomes, the data from this study did not support the use of DET-GQE + PQE compared with SET-GQE in patients younger than 35 years. Additionally, considering the robust decrease in the LBR following single cleavage-stage embryo transfer in patients aged 35 years and older, we highlighted that single blastocyst-stage embryo transfer, rather than single cleavage transfer, appeared to be a more promising option when SET-GQE was conducted on patients aged 35 years and older.

Growing evidence has shown that multiple gestations after IVF/ICSI are associated with a significantly greater risk of neonatal and obstetric complications [3, 24]. In this study, we showed that DET resulted in many multiple live births, regardless of the embryo stage. Therefore, to promote a healthy singleton gestation and reduce the number of multiple gestations while maximizing the CLBR, eSET should be strongly recommended. Interestingly, according to the data in our study, the addition of a second PQE to a GQE transfer did not diminish the likelihood of live birth. In other words, these results did not support the hypothesis that a PQE might decrease the chance of live birth via negative embryo-endometrial crosstalk [11]. Conversely, the addition of a PQE resulted in a significantly greater LBR and an absolute increase in MBR in patients who underwent blastocyst-stage embryo transfer, with the MBR ranging from 1%—20.6%. As a consequence, higher PBRs and more low-birth-weight babies were observed when transferring a second PQE. Previous studies have reported conflicting results when comparing IVF/ICSI outcomes of SET with DET in fresh or FET cycles. According to the results of a recent systematic review and meta-analysis, double ET with a PQE in addition to a GQE does not result in increased or decreased CPR or LBR compared with single ET with a GQE but leads to a greater MBR [25]. One study concerning the addition of a PQE in fresh or frozen-thawed blastocyst-stage embryo transfers showed that DET with one GQE plus one PQE resulted in a decrease in the LBR, but the difference compared to the result of SET with one GQE was not significant [26]. In contrast, other studies have indicated that the addition of a PQE does not have a detrimental effect on the GQE and results in a small increase in live births, except for a marked increase in the likelihood of multiple gestations [13, 14]. According to a recent large sample size study, Zhu et al. suggested that transferring a PQE along with a GQE did not significantly affect live births but the MBR was greater from GQEs only in frozen-thawed blastocyst-stage embryo transfer cycles [24]. However, in another recent study, Wang et al. [14] reported that the addition of a lower-quality blastocyst resulted in increases in both live births and multiple gestations. Consistent with the findings of previous studies, our findings suggested that transferring a second PQE did not diminish the likelihood of live birth, regardless of the embryo stage. Nevertheless, the addition of a PQE contributed to both live birth and multiple births in patients who underwent blastocyst-stage embryo transfer. To our knowledge, few studies have compared neonatal outcomes between SET with a GQE and DET with a GQE and a PQE. In this study, transferring a second PQE resulted in an inherent risk of adverse neonatal outcomes associated with multiple gestations in patients who underwent blastocyst-stage embryo transfer cycles rather than cleavage-stage cycles, which is in accordance with the findings highlighted by a previous retrospective study [24]. Thus, the benefits and risks of double blastocyst-stage embryo transfer are balanced.

In clinical practice, the physicians’ suggested number of transferred embryos is usually based on the age of the patient. Limiting the number of embryos transferred needs to be balanced with the risk of decreasing the overall pregnancy rate. Thus, physicians generally tend to consider transferring two embryos when patients have unfavorable pregnancy prognoses, such as older age, decreased embryo quality or no previous live birth after an IVF cycle. According to the newly published guidance on the number of embryos to be transferred by the American Society for Reproductive Medicine (ASRM), patients younger than 35 years of age should be strongly encouraged to receive a single-embryo transfer, regardless of the embryo stage [2]. In addition, given the anticipated age-related decline in fertility, the increased incidence of disorders that impair fertility, and an increased risk of pregnancy loss, women 35 years and older should receive an expedited evaluation and undergo ART treatment after 6 months of failed attempts to conceive or earlier [27]. Thus, we stratified our analysis by age (35 years) to evaluate the impact of age on IVF outcomes. As mentioned above, in patients younger than 35 years of age, we noticed an increase of 11.0% in LBR, while having a robust 24.2% increase in MBR for blastocyst-stage embryo transfer. Moreover, transferring a second PQE increased the risk of adverse neonatal outcomes associated with multiple gestations, as indicated by a higher PBR and lower gestational age and birth weight. Patients 35 years and older had only a small benefit from the transfer of a second poor-quality blastocyst-stage embryo, as there was no obvious increase in the LBR, whereas the MBR increased by 8.3%. These findings are in line with previous data showing the benefit of SET in patients younger than 35 years [13, 28]. Interestingly, the study by Wang et al. [14] showed that in patients aged < 35 years, the multiple pregnancy rate (MPR) was significantly greater in the GQE + PQE group than in the single GQE group, with no significant differences in the LBR. However, for patients aged 35 years and older, the MPR and LBR were significantly greater in the GQE + PQE group than in the single GQE group. Thus, additional studies involving subgroup analyses based on maternal age are needed.

To our knowledge, few studies have evaluated the impact of the addition of a PQE in frozen-thawed cleavage-stage embryo transfer. Recently, Zhu et al. reported an increased LBR after DET with a GQE plus a PQE compared with SET with a GQE for vitrified cleavage-stage embryo transfer, but the difference was not significant for blastocyst-stage embryo transfer during the first FET treatment [24]. In our study, regardless of age, we did not observe a significant increase in the LBR with the addition of a second PQE in patients who underwent cleavage-stage embryo transfer. Moreover, the addition of a PQE during frozen-thawed Day 3 DET significantly increased the MBR by 18.4% in patients younger than 35 years. This result was consistent with that of a previous study conducted by Berkhout et al. [7] on Day 3 of fresh DET. However, the LBR decreased dramatically to 19.6% following single cleavage-stage embryo transfer in women 35 years and older, but no significant difference was observed, partly due to the small sample size in this study. Given the conflicting results of the existing research, further studies concerning the pregnancy and neonatal outcomes of FETs on Day 3 in women 35 years and older are still needed. In addition, single blastocyst-stage embryo transfer, rather than single cleavage-stage embryo transfer, appeared to be a more promising option and did not compromise the LBR [29]. These data support the findings highlighted by a previous randomized trial that revealed higher rates of ongoing pregnancy following blastocyst-stage embryo transfer than after cleavage-stage embryo transfer in women 35 years of age and older [30].

The main strength of our study is that PSM was conducted to control for potential confounders that could have influenced the results, as there were significant differences in the baseline characteristics of the overall groups. PSM provides an approach to mimic random assignment as an RCT and is superior to conventional regression-based methods in a real-world observational study [23]. Additionally, comparisons were not only performed for overall groups but also for female patients stratified by age. Moreover, in this study, we explored the relationship between neonatal outcomes and the number and quality of embryos transferred in patients undergoing FET cycles, which has seldom been mentioned in other studies. Unfortunately, our study has several limitations that need to be taken into consideration. First, our study is limited by its retrospective observational design. Although PSM was performed to evaluate the effects of additional PQE with GQE on IVF/ICSI outcomes, the sample size decreased after PS, and the loss of unmatched cases might have unforeseen effects. Moreover, the sample size of patients 35 years and older who underwent cleavage-stage embryo transfer was small, so it is not easy to determine the best method of embryo transfer for these patients. Therefore, further large randomized clinical trials and experimental in vitro studies are still needed to determine whether adding a PQE truly affects endometrial signaling and clinical outcomes.

## Conclusions

Overall, transferring an additional PQE along with a GQE did not have an obvious benefit on clinical outcomes, as the difference in the LBR was not significant between the group with the added PQE and the group that just had a GQE. However, the MBR was increased for cleavage-stage embryo transfer. In addition, for patients who underwent blastocyst-stage embryo transfer, DET + GQE + PQE resulted in an increase in both the LBR and MBR, which may lead to adverse neonatal outcomes. Thus, the benefits and risks of double blastocyst-stage embryo transfer should be balanced. In addition, the current study provides novel information regarding the suggested number of embryos to transfer based on patient age. In patients younger than 35 years, SET-GQE resulted in satisfactory LBR either in the cleavage-stage embryo transfer group or blastocyst-stage embryo transfer group, while DET-GQE + PQE resulted in a dramatically increased MBR. Thus, in order to minimize the risk of multiple live births and adverse neonatal outcomes, the data from this study did not support the use of DET-GQE + PQE rather than SET-GQE in patients younger than 35 years, regardless of the embryo stage. Considering the dramatically decreased LBR following single cleavage-stage embryo transfer in women 35 years and older, single blastocyst-stage embryo transfer, rather than single cleavage-stage embryo transfer, appears to be a more promising approach without compromising the LBR.

## Data Availability

The datasets used and/or analysed during the current study are available from the corresponding author on reasonable request.

## Abbreviations

PQE: Poor quality embryo
GQE: Good quality embryo
SET: Single embryo transfer
DET: Double embryo transfer
CPR: Clinical pregnancy rate
LBR: Live birth rate
MBR: Multiple birth rate
PBR: Preterm birth rate
IVF/ICSI: In vitro fertilization/ intracytoplasmic sperm injection
ART: Assisted reproductive technology
FET: Frozen thawed embryo transfer
PSM: Propensity score matching
GEE: Generalized estimating equations
ICM: Inner cell mass
TE: Trophectoderm
OPU: Oocyte pickup
TS: Thawing solution
HRT: Hormone replacement treatment
NC: Natural cycle
GnRHa: Gonadotropin-releasing hormone agonist
ET: Embryo transfer
SD: Standard deviation
OR: Odds ratios
CI: Confdence intervals
BMI: Body mass index
Gn: Gonadotropins
AMH: Antimullerian hormone
AFC: Antral follicle count

## References

[1] Practice Committee of the American Society for Reproductive Medicine. Practice Committee of the Society for Assisted Reproductive Technology. Guidance on the limits to the number of embryos to transfer: a committee opinion. Fertil Steril. 2017;107(4):901–3. 10.1016/j.fertnstert.2017.02.107 28292618

[2] Practice Committee of the American Society for Reproductive Medicine and the Practice Committee for the Society for Assisted Reproductive Technologies. Guidance on the limits to the number of embryos to transfer: a committee opinion. Fertil Steril. 2021;116(3):651–4. 10.1016/j.fertnstert.2021.06.050 34330423

[3] Santana D, Cecatti J, Surita F, Silveira C, Costa M, Souza J (2016). Twin pregnancy and severe maternal outcomes: The World Health Organization multicountry survey on maternal and newborn health. Obstet Gynecol.

[4] Multiple gestation pregnancy (2000). The ESHRE capri workshop group. Hum Reprod (Oxford, England).

[5] Eapen A, Ryan G, Ten Eyck P, Van Voorhis B (2020). Current evidence supporting a goal of singletons: a review of maternal and perinatal outcomes associated with twin versus singleton pregnancies after in vitro fertilization and intracytoplasmic sperm injection. Fertil Steril.

[6] Oron G, Son W, Buckett W, Tulandi T, Holzer H (2014). The association between embryo quality and perinatal outcome of singletons born after single embryo transfers: a pilot study. Hum Reprod (Oxford, England).

[7] Berkhout R, Vergouw C, van Wely M, de Melker A, Schats R, Repping S (2017). The addition of a low-quality embryo as part of a fresh day 3 double embryo transfer does not improve ongoing pregnancy rates. Human Reprod Open.

[8] Kamath M, Mascarenhas M, Kirubakaran R, Bhattacharya S (2020). Number of embryos for transfer following in vitro fertilisation or intra-cytoplasmic sperm injection. Cochrane Database Syst Rev.

[9] Clua E, Tur R, Coroleu B, Rodríguez I, Boada M, Gómez M (2015). Is it justified to transfer two embryos in oocyte donation? A pilot randomized clinical trial. Reprod Biomed Online.

[10] Ma S, Peng Y, Hu L, Wang X, Xiong Y, Tang Y (2022). Comparisons of benefits and risks of single embryo transfer versus double embryo transfer: a systematic review and meta-analysis. Reprod Biol Endocrinol.

[11] Macklon N, Brosens J (2014). The human endometrium as a sensor of embryo quality. Biol Reprod.

[12] Ohgi S, Taga Y, Anakubo H, Kurata Y, Hatakeyama S, Yanaihara A (2018). Morphologically poor blastocysts could affect the implantation rate of a morphologically good blastocyst during a double-blastocyst transfer for patients who have experienced repeated implantation failures. Reprod Med Biol.

[13] Hill M, Eubanks A, Csokmay J, Christy A, Jahandideh S, DeCherney A (2020). Is transferring a lower-quality embryo with a good-quality blastocyst detrimental to the likelihood of live birth?. Fertil Steril.

[14] Wang W, Cai J, Liu L, Xu Y, Liu Z, Chen J (2020). Does the transfer of a poor quality embryo with a good quality embryo benefit poor prognosis patients?. Reprod Biol Endocrinol.

[15] Aldemir O, Ozelci R, Baser E, Kaplanoglu I, Dilbaz S, Dilbaz B (2020). Impact of transferring a poor quality embryo along with a good quality embryo on pregnancy outcomes in IVF/ICSI cycles: a retrospective study. Geburtshilfe Frauenheilkd.

[16] Zeng C, Shang J, Jin AM, Wu PL, Li X, Xue Q (2019). The effect of luteal GnRH antagonist on moderate and severe early ovarian hyperstimulation syndrome during in vitro fertilization treatment: a prospective cohort study. Arch Gynecol Obstet.

[17] Li X, Zeng C, Shang J, Wang S, Gao XL, Xue Q (2019). Association between serum estradiol level on the human chorionic gonadotrophin administration day and clinical outcome. Chin Med J.

[18] Alpha Scientists in Reproductive Medicine and ESHRE Special Interest Group of Embryology. The Istanbul consensus workshop on embryo assessment: proceedings of an expert meeting. Human Reprod (Oxford, England). 2011;26(6):1270–83. Epub 2011/04/20. 10.1093/humrep/der037 21502182

[19] Gardner D, Schoolcraft W (1999). Culture and transfer of human blastocysts. Curr Opin Obstet Gynecol.

[20] Kuwayama M, Vajta G, Kato O, Leibo SP (2005). Highly efficient vitrification method for cryopreservation of human oocytes. Reprod Biomed Online.

[21] Parmegiani L, Beilby KH, Arnone A, Bernardi S, Maccarini AM, Nardi E (2018). Testing the efficacy and efficiency of a single "universal warming protocol" for vitrified human embryos: prospective randomized controlled trial and retrospective longitudinal cohort study. J Assist Reprod Genet.

[22] Li X, Zeng C, Wu P, Shang J, Xue Q (2023). Predictive value of D-dimer in patients with unexplained recurrent implantation failure during freeze-thaw embryo transfer cycles. Am J Reprod Immunol.

[23] Agoritsas T, Merglen A, Shah ND, O'Donnell M, Guyatt GH (2017). Adjusted analyses in studies addressing therapy and harm: users' guides to the medical literature. JAMA.

[24] Zhu Q, Lin J, Gao H, Wang N, Wang B, Wang Y (2020). The association between embryo quality, number of transferred embryos and live birth rate after vitrified cleavage-stage embryos and blastocyst transfer. Front Physiol.

[25] Xiao Y, Wang X, Gui T, Tao T, Xiong W (2022). Transfer of a poor-quality along with a good-quality embryo on in vitro fertilization/intracytoplasmic sperm injection-embryo transfer clinical outcomes: a systematic review and meta-analysis. Fertil Steril.

[26] Dobson SJA, Lao MT, Michael E, Varghese AC, Jayaprakasan K (2018). Effect of transfer of a poor quality embryo along with a top quality embryo on the outcome during fresh and frozen in vitro fertilization cycles. Fertil Steril.

[27] American College of Obstetricians and Gynecologists Committee on Gynecologic Practice and Practice Committee. Female age-related fertility decline. Committee Opinion No. 589. Fertil Steril. 2014;101(3):633–4. Epub 2014/02/25. 10.1016/j.fertnstert.2013.12.032 24559617

[28] Wang Z, Zhu H, Tong X, Jiang L, Wei Q, Zhang S (2022). Clinical outcomes after elective double-embryo transfer in frozen cycles for women of advanced maternal age: A retrospective cohort study. Medicine.

[29] Park D, Kim J, Chang E, Lee W, Yoon T, Lyu S (2019). Strategies in the transfer of varying grades of vitrified-warmed blastocysts in women aged over 35 years: A propensity-matched analysis. J Obstet Gynaecol Res.

[30] Fernández-Shaw S, Cercas R, Braña C, Villas C, Pons I (2015). Ongoing and cumulative pregnancy rate after cleavage-stage versus blastocyst-stage embryo transfer using vitrification for cryopreservation: impact of age on the results. J Assist Reprod Genet.

